# Peripapillary Microvascularization Analysis Using Swept-Source Optical Coherence Tomography Angiography in Optic Chiasmal Compression

**DOI:** 10.1155/2021/5531959

**Published:** 2021-09-04

**Authors:** Inès Ben Ghezala, Déa Haddad, Julie Blanc, Cyril Meillon, Rachid Madkouri, François Borsotti, Alain M. Bron, Catherine Creuzot-Garcher

**Affiliations:** ^1^Department of Ophthalmology, University Hospital, Dijon, France; ^2^Department of Neurosurgery, University Hospital, Dijon, France; ^3^Taste and Food Science Centre, AgroSup Dijon, CNRS, INRAE, Bourgogne Franche-Comté University, F-21000 Dijon, France

## Abstract

**Purpose:**

To evaluate the vessel density (VD) of the radial peripapillary capillary (RPC) network using swept-source optical coherence tomography angiography (SS-OCTA) “en face” images of eyes with chiasmal compression caused by brain tumors before and after decompressive surgery compared with healthy controls.

**Methods:**

A cross-sectional study was conducted in 12 patients with chiasmal compression confirmed by neuroimaging. Sixteen healthy participants were also included. All patients with chiasmal compression underwent a neuro-ophthalmological examination one week before and 6 months after brain surgery, including static automated perimetry as well as measurement of the thickness of the retinal nerve fiber layer (RNFL) and the ganglion cell complex (GCC) with spectral-domain optical coherence tomography (SD-OCT). Based on this neuro-ophthalmological examination, the presence of an optic neuropathy (ON) was evaluated. Peripapillary VD was obtained in four sectors on a 6 × 6 mm SS-OCTA image using the Cirrus Plex Elite 9000.

**Results:**

Baseline average VD was significantly lower in patients with chiasmal compression and ON than in controls (median: 55.62; interquartile range (IQR): 2.96 vs. 58.53; IQR: 2.02; *p*=0.003). This decrease was also found in the temporal, superior, and nasal sectors. Average postoperative VD was decreased in patients with chiasmal compression compared with average preoperative VD (median: 56.16; IQR: 4.07 vs. 57.48; IQR: 3.83; *p*=0.004). Preoperative VD was significantly correlated with RNFL, GCC thickness, and visual field defects.

**Conclusions:**

The VD of the RPC network was decreased in chiasmal compressive ON, and it was further decreased at 6 months after decompressive surgery.

## 1. Introduction

Various brain tumors can cause chiasmal compression: adenomas of the pituitary gland, craniopharyngiomas, pinealomas, Rathke cleft cysts, and meningiomas. Chiasmal compression predominantly affects the crossed nerve fibers associated with the nasal hemiretina, leaving uncrossed nerve fibers (coming from the temporal hemiretina) relatively well-preserved [1, 2]. This compression increases the risk of damage to the axons of retinal ganglion cells, leading to deterioration of visual function, particularly of the visual field (VF). It is traditionally diagnosed by the presence of a characteristic bitemporal hemianopsia on the VF [3].

Optical coherence tomography (OCT) has been widely used to assess the morphological alterations of the retina and the peripapillary retinal nerve fiber layer (RNFL) in compressive optic neuropathy (ON). Previous studies have documented that peripapillary and macular retinal thickness, particularly RNFL, ganglion cell layer, and inner plexiform layer, were correlated with the location and the severity of the VF defects [4–7]. Nasal retinal thickness is especially strongly correlated with temporal VF [7]. Measurement of RNFL thickness was also used to predict visual recovery after decompressive surgery [6, 8, 9]. Macular ganglion cell complex (GCC) analysis has been shown to be especially useful in the early detection of chiasmal compression without VF defects, and it might be more sensitive than RNFL thickness [10–14].

Recent advances in optical coherence tomography angiography (OCTA) have led to the identification of several microvascular alterations in various ONs and retinal diseases. This recently developed imaging technique can noninvasively visualize the retinal and peripapillary microcirculation without dye injection [15, 16]. Retinal vascularization is organized into four vascular plexuses, the superficial capillary plexus (SCP), which then anastomoses and generates the intermediate and deep capillary plexus (ICP and DCP, respectively) on either side of the inner nuclear layer, a superficial plexus in the RNFL around the optic nerve head, and the radial peripapillary capillary (RPC) plexus [17]. This RPC network cannot be clearly visualized with fluorescein angiography, unlike with OCTA images [18].

Recent studies have analyzed the contribution of OCTA in neuropathic disorders; microvascular changes were found in glaucoma [19–21], ischemic ON [22–24], and inflammatory ON [25–27]. A significant correlation was found between RPC density, RNFL thickness, and severity of visual function defects in glaucomatous eyes [20, 21]. Even in healthy eyes, a correlation between RNFL thickness and RPC density has been reported [28].

Nevertheless, to date, few studies have investigated the microvascular changes in eyes with chiasmal compression using OCTA [29–32]. Circumpapillary and macular vessel density have been shown to be lower in eyes with chiasmal compression than in healthy control eyes, especially in the nasal sectors [29, 31]. Circumpapillary and macular vessel density were correlated with RNFL thickness, GCC analysis, and VF defects [29].

These findings stimulated the hypothesis that a change in peripapillary microvascularization may be associated with a compressive ON and might be a predictive factor of postoperative VF recovery. Therefore, the present study aimed to quantify the VD of the RPC network using swept-source optical coherence tomography angiography (SS-OCTA) “en face” images from patients with chiasmal compression tumors and compare them with healthy control volunteers. We also evaluated the kinetics of VD after decompressive brain surgery and the correlation with other ocular metrics, such as VF defects, RNFL thickness, and GCC analysis.

## 2. Materials and Methods

### 2.1. Study Participants

We conducted a prospective study in the Department of Ophthalmology at the Dijon University Hospital (France) between April 1^st^, 2019, and March 1^st^, 2020. This study included consecutive patients with chiasmal compression confirmed by neuroimaging and healthy controls. The protocol was approved by the regional ethics committee and followed the tenets of the Declaration of Helsinki. All patients gave their written informed consent for the examination before enrolment. Patients were excluded if they had any retinal disease or optic nerve disease other than compressive ON. They were also excluded if they had a history of diabetes, cardiovascular diseases, or any other diseases that might affect the retina or the optic nerve. Concerning the brain lesion, patients were excluded if they had undergone any previous radiotherapy, surgery, or medical treatments. All patients had magnetic resonance imaging (MRI) of the brain that confirmed a lesion compressing the optic chiasm. All patients underwent decompressive brain surgery, a transsphenoidal tumor resection, at the Department of Neurosurgery of Dijon University Hospital. The control group consisted of healthy volunteers. Controls with a self-reported eye disease or with a history of cardiovascular or neurological disease were excluded.

### 2.2. Clinical Evaluation

**Figure 1:**
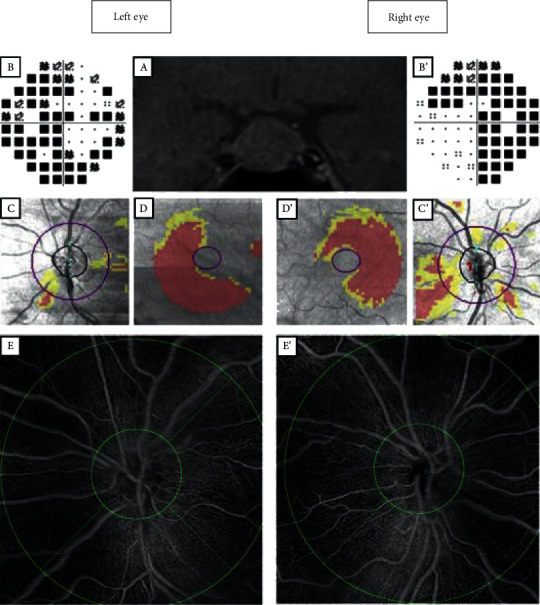
Multimodal analysis of a patient with a large pituitary adenoma with bilateral optic neuropathy. (A) Brain T1 magnetic resonance imaging showing a large pituitary adenoma with a mass effect on the optic chiasm. (B, B′) Bitemporal hemianopsia on the Humphrey visual field (30–2). (C, C′) Retinal nerve fiber layer thickness map. (D, D′) Ganglion cell complex analysis. (E, E′) “En face” optical coherence tomography angiography, 6 × 6 mm, with early treatment diabetic retinopathy study grid.

All patients and healthy controls underwent a complete neuro-ophthalmological evaluation, including measurement of the best corrected visual acuity with a Monoyer chart, dilated fundoscopic examination, intraocular pressure measurement with a noncontact tonometer (Tonoref II, Nidek, Gamagori, Japan), globe axial length measurement using the IOL master 500 (Carl Zeiss Meditec, Jena, Germany), color vision testing with Ishihara plates, and VF and SD-OCT before and after surgery. The VF was assessed with an automated perimetry with the 30-2 Swedish interactive thresholding algorithm (SITA) standard strategy (Humphrey field analyzer, Carl Zeiss Meditec, Dublin, CA, USA). The reliability criteria were fixation loss of ≤20%, false-positive results of ≤15%, and false-negative results of ≤15%. The mean deviation (MD) and pattern standard deviation (PSD) were used for the analysis. The SD-OCT analysis was performed with the Cirrus HD-OCT 5000 (Carl Zeiss Meditec, Dublin, CA, USA). The RNFL thickness was determined using the 200 × 200 protocol optical disc cube with Cirrus software (Carl Zeiss Meditec, Dublin, CA, USA). This protocol provides an average thickness of the RNFL and the thickness in four sectors (temporal, superior, nasal, and inferior). For the GCC analysis, the thickness of the ganglion cell layer and the inner plexiform layer were automatically evaluated. Based on clinical examination, compatible VF defects, GCC, and RNFL loss on SD-OCT, we defined two subgroups of patients with chiasmal compression: patients with and without ON (ON+ and ON–, respectively). Preoperative assessments were performed one week before surgery, and postoperative visits occurred 6 months after surgery.  Figure 1 illustrates the multimodal analysis of a patient with chiasmal compression ON at the time of diagnosis.

### 2.3. OCTA Imaging

The SS-OCTA images were acquired with the Cirrus Plex Elite 9000 (Carl Zeiss Meditec, Jena, Germany), which uses a swept laser source with a central wavelength between 1040 nm and 1060 nm and a rate of 100,000 A-scans per second. For all study participants, the SS-OCTA examination was performed under mydriasis obtained with one drop of tropicamide 0.5% (Théa, Clermont-Ferrand, France). Patients underwent one imaging session including a 6 × 6 mm scan centered on the optic nerve head. We used an active eye tracker to minimize motion artefacts. Eyes with poor signal strength (<6/10) or significant artefacts on the perfusion map were excluded from the analysis.

The software automatically performed segmentation of the inner limiting membrane and the outer boundary of the RNFL to isolate the RPC vasculature “en face.” Anonymized data files were downloaded from the Cirrus Plex Elite 9000 (Carl Zeiss Meditec, Jena, Germany) and uploaded to the advanced retina imaging (ARI) network portal. Image processing was performed with the peripapillary nerve fiber layer microvasculature density algorithm developed by Carl Zeiss Meditec (version 0.7).

### 2.4. Radial Peripapillary Capillary Analysis

Vessel density was defined as the percentage of the area occupied by vessels showing blood flow in a given region. The software automatically defined the center of the optic disc and identified four sectors (temporal, superior, nasal, and inferior) within two rings of 2 mm as the inner diameter and 6 mm as the outer diameter (Figure 1). Vessel density was provided in the four sectors as well as the average of these four sectors. The result was a percentage ranging from 0% (no perfusion) to 100% (fully perfused). The area occupied by large retinal vessels was not included in these calculations.

### 2.5. Statistical Analysis

Continuous variables were tested for normality (Shapiro–Wilk test). Continuous variables with a normal distribution are expressed as mean (standard deviation, SD) and those with a nonnormal distribution are expressed as median (interquartile range, IQR). Categorical variables are summarized as numbers and percentages. Generalized estimating equation (GEE) regression models were used to consider intraindividual correlations between the two eyes to compare RNFL thickness, GCC thickness, visual field, and vessel density between patients and controls and between preoperative and postoperative values in patients. Spearman's correlation coefficients were used to determine the relationship between peripapillary VD and traditional metrics such as RNFL and GCC thickness and VF, PSD, and MD in eyes with chiasmal compression. A *p* value of less than 0.05 was considered statistically significant. All statistical analyses were performed using SAS software (version 9.4; SAS Institute, Inc., Cary, NC, USA).

## 3. Results

**Figure 2:**
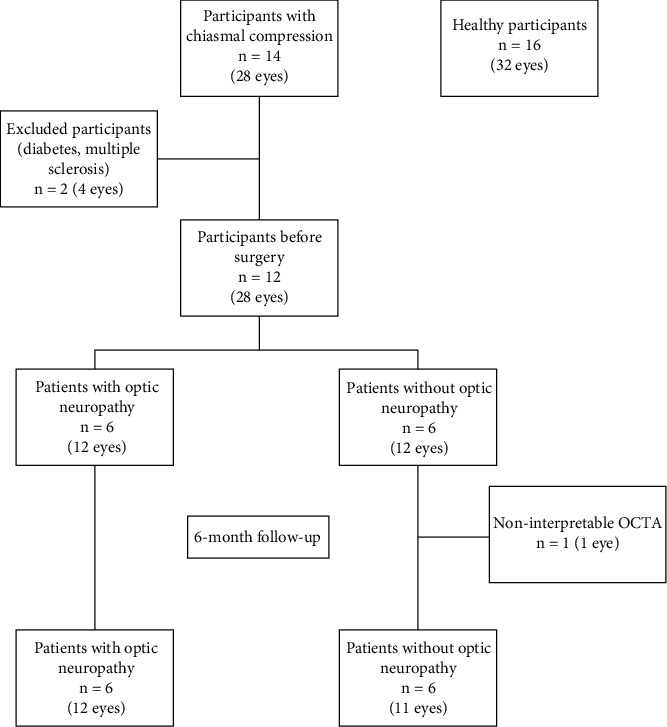
Flowchart of eligible and excluded patients and healthy participants.

The study included 14 consecutive patients with chiasmal compression and 16 healthy participants. Two patients with chiasmal compression were excluded owing to coexisting diabetes and multiple sclerosis. A total of 32 eyes from 16 healthy participants (group 1) and 24 eyes from 12 patients with chiasmal compression (group 2) were included in the analysis. Brain MRI revealed the following diagnoses: ten pituitary adenomas, one intrasellar arachnoid cyst, and one meningioma. An ON was found in 12 eyes from six patients (five pituitary adenomas and one meningioma). One eye of a patient with ON was excluded after neurosurgery due to poor SS-OCTA quality related to postoperative oculomotor paralysis, and the other eye was retained for analysis (Figure 2).

Tables 1 and 2 summarize the demographic as well as the structural and functional characteristics of the patients and healthy controls.

Table 3 presents the results of the comparison of VD across sectors between healthy controls and patients with chiasmal compression.

Baseline average VD was significantly lower in patients with chiasmal compression ON+ than in controls (*p*=0.003). Based on a sector comparison, VD was significantly reduced in patients with chiasmal compression ON+ in the temporal, superior, and nasal sectors, compared with healthy controls (*p*=0.005, *p*=0.02, and *p*=0.03, respectively).

### 3.1. Vessel Density of the Radial Peripapillary Capillary 6 Months after Surgery

Table 4 lists the changes in VD in the RPC network 6 months after decompressive surgery in all sectors.

VD was reduced postoperatively compared with preoperatively in patients with chiasmal compression (median: 56.16; IQR: 4.07 vs. 57.48; IQR: 3.83; *p*=0.004). This significant reduction was also found in the temporal, superior, and inferior sectors.

### 3.2. Correlation between Vessel Density and Structural and Functional Metrics

Table 5 displays the Spearman correlation coefficients of VD before surgery with RNFL, GCC, and VF defects. The relationship between VD and RNFL thickness was analyzed in all patients with chiasmal compression in the different sectors segmented. A correlation was found between RNFL thickness and average, temporal, superior, and inferior VD (*p* < 0.001, *p* < 0.001, *p*=0.004, and *p*=0.005, respectively).

## 4. Discussion

In this study, we investigated peripapillary retinal microvascularization in patients with chiasmal compression before and after decompressive surgery and compared these patients with healthy volunteers. Average, temporal, superior, and nasal VD was significantly lower in patients with chiasmal compression ON+ than in controls. Interestingly, VD was higher in patients with chiasmal compression ON- than in controls.

In the literature, it has been established that the vascular network plays a primary role in many ONs [27, 33]. However, the possibility of a primary vascular component in compressive ON is unlikely. The reduction in VD could be secondary to neural degeneration of the retina. It has already been established that chiasmal compression affects the nerve fibers in the nasal hemiretina [1, 2]. This compression resulted in optic atrophy characterized clinically by band atrophy in the fundus [2], associated with a decrease in RNFL and GCC thicknesses [14, 34]. Functionally, it was defined by bitemporal hemianopsia on the VF [3]. Therefore, the neuronal and axonal loss could reduce the metabolic activity in the inner layers of the retina, leading to a decrease in the demand for oxygen and blood with a constriction of precapillary arterioles [35]. Thus, this reduction could induce a regression of the superficial peripapillary capillary network, particularly pronounced in the nasal sector corresponding to axonal loss. This hypothesis of neurovascular metabolic feedback between loss of neurons and reduction in vascular perfusion has already been discussed but remains under debate [31, 36]. However, the hypothesis appears to be consistent with our results since VD reduction was strongly correlated with RNFL thickness reduction.

We also investigated the changes in VD after decompressive surgery. The 6-month postoperative follow-up of patients demonstrated a significant decrease in average VD in patients with chiasmal compression (*p*=0.004). We might have expected an increase in VD postoperatively after the removal of axonal compression, but this was not observed in the present case series, we found the opposite. Postoperative disc swelling may have gone undetected and could lead to changes in VD, as was previously described in other studies [37, 38].

Regarding the relationship between VD in SS-OCTA and other ocular metrics, a fairly positive correlation between average VD and RNFL thickness was found for patients with chiasmal compression (*r* = 0.69, *p* < 0.001). This correlation was found with pre- and postoperative RNFL thickness and has already been shown in other studies on compressive ON [29] and in glaucomatous eyes [19]. Interestingly, we found a significant correlation between preoperative VD and preoperative VF, like Suzuki et al. [29]. Nevertheless, we did not find any correlation between preoperative VD and postoperative VF.

Our results are consistent with previous studies that have also demonstrated a decrease in peripapillary and macular VD in ON secondary to chiasmal compression [29]. Peripapillary and macular VD have been studied in patients with pituitary adenomas, and a significant decrease in VD in the peripapillary and macula SCP has been shown [39]. To our knowledge, only a few studies have investigated the impact of decompressive surgery on VD. Lee et al. also found a decrease in peripapillary VD after surgery [31]. One study found a correlation between preoperative peripapillary VD and postoperative VF, suggesting this could be a prognostic factor [32]. In our study, although peripapillary VD decreased after surgery, patients had a relatively well-preserved postoperative VF (mean deviation −2.8 dB ± 5.5 for patients who had chiasmal compressive ON) and we did not find any correlation between average preoperative VD and postoperative VF (*p*=0.54 for VF mean deviation). Interestingly, decreased retinal perfusion was also demonstrated after treatment of optic neuritis with good visual acuity recovery [40].

We acknowledge several limitations to this study. First, this is a small case series within a single center, which limits its external validity. Second, medical history was mostly self-reported, and we did not carry out any medical assessment or any blood tests to formally exclude vascular diseases in healthy participants. Third, we encountered artefacts with the acquisition of SS-OCTA images, for example, artefacts during OCTA image acquisition, including motion artefacts, and during image treatment such as automated segmentation failure [41]. However, projection artefacts were not expected to affect the measurements in this study given that the microvascularization within the RNFL is the most anterior retinal vascularization. Besides, only eyes with good SS-OCTA signal strength (≥7/10) and without any significant artefact on the perfusion map were included. Fourth, the quantification grid was automatically centered on the optic disc for VD measurement. It is possible that this detection failed and resulted in inaccurate VD quantification. We therefore checked the correct location of the optic disc on the OCTA acquisitions. Fifth, we could not rule out the possibility of undetected microswelling in the optic disc affecting the assessment of VD. Sixth, automatic quantification using OCTA software has demonstrated good repeatability and reproducibility [42–44]. However, the repeatability and reproducibility of this measurement in SS-OCTA with the Cirrus Plex Elite 9000 device (Carl Zeiss Meditec, Jena, Germany) have not been studied. Since the same measurements using different devices are not comparable, further study should evaluate the repeatability and reproducibility of this measurement with the Cirrus Plex Elite 9000 device (Carl Zeiss Meditec, Jena, Germany) [45].

In conclusion, the VD of the RPC network was decreased in chiasmal compressive ON. This decrease was correlated with other optic nerve metrics. The effect of decompressive surgery may potentially play a role in induced and additional decrease in the final VD reduction.

## Figures and Tables

**Table 1:** Demographic characteristics of study participants.

	Healthy controls (*N* = 16)	Patients with chiasmal compression (*N* = 12)	*p* value
Eyes	32	24	
Sex, female	13 (81.2)	8 (66.6)	0.51^*∗*^
Age, years	46.7 ± 14.1	53.1 ± 14.5	0.10^*∗∗*^
IOP, mmHG	15.9 ± 2.4	17.2 ± 1.3	0.11^*∗∗*^
Axial length, mm	23.8 ± 0.7	23.9 ± 1.2	0.59^*∗∗*^

Continuous variables are displayed as mean ± standard deviation. Categorical variables are displayed as number (percentage). IOP = intraocular pressure. ^*∗*^Fisher's exact test ^*∗∗*^Student's *t*-test.

**Table 2:** Structural and functional characteristics of patients with chiasmal compression.

	Patients with chiasmal compression with ON			Patients with chiasmal compression without ON		
	Preoperative (*n* = 6) (12 eyes)	Postoperative (*n* = 6) (12 eyes)	*p* value^†^	Preoperative (*n* = 6) (12 eyes)	Postop (*n* = 6) (11 eyes)	*p* value^†^
*RNFL thickness, μm* ^*∗*^						
Average	76.4 ± 2.3	71.8 ± 3.0	**0.001**	90.7 ± 2.6	88.8 ± 2.9	0.42
Temporal	48.8 ± 2.4	47.0 ± 3.3	0.06	66.8 ± 4.3	64.7 ± 1.8	0.52
Superior	97.3 ± 3.8	88.0 ± 4.8	**<0.001**	108.2 ± 5.6	105.7 ± 6.3	0.60
Nasal	62.6 ± 3.6	58.0 ± 2.1	**0.02**	70.6 ± 3.3	70.5 ± 3.9	0.96
Inferior	98.1 ± 5.4	94.4 ± 7.3	0.21	117.2 ± 1.4	117.1 ± 2.3	0.96

*GCC thickness, μm* ^*∗*^		63.8 ± 3.3				
Average	63.8 ± 3.2	63.8 ± 3.3	0.89	78.2 ± 4.3	77.1 ± 3.1	0.62

*Visual field, dB* ^*∗∗*^						
Mean deviation	−14.3 ± 13.0	-2.8 ± 5.5	**0.003**	−2.6 ± 5.6	−3.7 ± 5.4	0.32
Pattern standard deviation	8.7 ± 7.1	3.4 ± 7.2	**0.04**	3.8 ± 2.6	3.0 ± 3.1	0.68

^*∗*^Variables are displayed as mean ± standard deviation. ^*∗∗*^Variables are displayed as median ± interquartile range.^†^Estimated using generalized estimating equation regression models. ON = optic neuropathy, RNFL = retinal nerve fiber layer, GCC = ganglion cell complex. No significant differences were found in gender and age between patients with chiasmal compression and healthy controls. No disc swelling was detected in the group of patients with chiasmal compression on fundus examination. In total, 13 eyes (54.2%) had acquired dyschromatopsia (eight in the group of patients ON+). Vessel density of the radial peripapillary capillary before surgery. Bold values represent statistical significance.

**Table 3:** Vessel density comparison between healthy controls and patients with chiasmal compression with and without optic neuropathy.

	Healthy controls (*N* = 16)	Patients with chiasmal compression with ON (*N* = 6)		Patients with chiasmal compression without ON (*N* = 6)	
	Median ± IQR	Median ± IQR	*p* value^*∗*^	Median ± IQR	*p* value^*∗*^
Average	58.53 ± 2.02	55.62 ± 2.96	**0.003**	59.44 ± 1.95	**0.01**
Temporal	59.05 ± 3.24	56.12 ± 3.63	**0.005**	61.83 ± 3.88	**<0.001**
Superior	57.91 ± 3.99	55.49 ± 3.72	**0.02**	58.01 ± 2.78	0.07
Nasal	56.49 ± 3.62	52.66 ± 7.36	**0.03**	57.12 ± 2.80	0.14
Inferior	59.73 ± 4.20	58.73 ± 3.99	0.15	61.08 ± 4.02	0.44

Variables are displayed as median ± interquartile range. ^*∗*^Estimated using generalized estimating equation regression models. ON = optic neuropathy. IQR = interquartile range.

**Table 4:** Pre- and postoperative vessel density comparison of patients with chiasmal compression with and without optic neuropathy.

	Patients with chiasmal compression (*n* = 12)		
	Preoperative	Postoperative	*p* value^*∗*^
Average	57.48 ± 3.83	56.16 ± 4.07	**0.004**
Temporal	58.30 ± 5.71	57.59 ± 6.95	**0.02**
Superior	57.02 ± 2.94	55.87 ± 6.46	**0.03**
Nasal	56.81 ± 5.52	55.21 ± 5.37	0.46
Inferior	59.94 ± 4.54	58.70 ± 4.40	**0.03**

Variables are displayed as median ± interquartile range. ^*∗*^Estimated using generalized estimating equation regression models. Bold values represent statistical significance.

**Table 5:** Spearman's correlation coefficient (*r* (*p* value)) of vessel density with pre- and postoperative structural and functional characteristics in patients with chiasmal compression.

Preoperative vessel density	Average	Temporal	Superior	Nasal	Inferior
*Preoperative*					
RNFL thickness	0.69 (**<0.001**)	0.75 (**<0.001**)	0.57 (**0.004**)	0.34 (0.10)	0.55 (**0.005**)
GCC thickness	0.60 (**0.002**)	NA	NA	NA	NA
VF mean deviation	0.64 (**0.001**)	NA	NA	NA	NA
VF pattern standard deviation	−0.79 (**<0.001**)	NA	NA	NA	NA

*Postoperative*					
RNFL thickness	0.80 (**<0.001**)	0.80 (**<0.001**)	0.71 (**<0.001**)	0.61 (**0.002**)	0.55 (**0.006**)
GCC thickness	0.58 **(0.003)**	NA	NA	NA	NA
VF mean deviation	0.13 (0.54)<	NA	NA	NA	NA
VF pattern standard deviation	−0.38 (0.07)	NA	NA	NA	NA

RNFL = retinal nerve fiber layer, GCC = ganglion cell complex, VF = visual field, NA = not applicable.

## Data Availability

Datasets analyzed during the current study are available from the corresponding author upon request.
